# Association between HDL-C and chronic pain: data from the NHANES database 2003–2004

**DOI:** 10.3389/fmed.2024.1340037

**Published:** 2024-03-11

**Authors:** Panpan Mi, Haoran Dong, Shengle Chen, Xuan Gao, Xu Cao, Yong Liu, Huijie Wang, Guofeng Fan

**Affiliations:** ^1^Department of Orthopedic, Hebei PetroChina Central Hospital, Langfang, China; ^2^Hospital of Stomatology Hebei Medical University, Shijiazhuang, China; ^3^Department of Endoscopy, Shijiazhuang Traditional Chinese Medicine Hospital, Shijiazhuang, China

**Keywords:** chronic pain, HDL-C, negative correlation, NHANES, cross-sectional study

## Abstract

**Objective:**

High-density lipoprotein cholesterol (HDL-C) has been reported to be associated with pain symptoms of various diseases, and its anti-inflammatory and antioxidant mediation is related to the pathogenesis of chronic pain. This study aims to evaluate the relationship between HDL-C levels and chronic pain in American adults.

**Methods:**

This cross-sectional study used data from American adults aged 20 and above during the 2003–2004 National Health and Nutrition Examination Survey (NHANES) cycle. Participants were divided into 4 groups based on HDL-C quartiles. We used chi-square tests and Student’s t-tests or Mann-Whitney U tests to analyze categorical variables and continuous variables to compare differences between groups. Multivariate logistic regression analysis was used to study the association between HDL-C levels and the risk of chronic pain. Likelihood ratio tests were used to assess interactions between subgroups, and sensitivity analyses were conducted.

**Results:**

Our final analysis included 4,688 participants, of which 733 (16.4%) had chronic pain. In the multivariate logistic regression model adjusted for covariates, there was a negative correlation between HDL-C levels and chronic pain. Specifically, for every 20 unit increase in HDL-C, the risk of chronic pain decreased by 26%. Compared with the lowest HDL-C quartile (< 43 mg/dL), the highest HDL-C quartile (≥ 64 mg/dL) was associated with a 24% reduction in the risk of chronic pain. No interaction factors affecting the relationship between HDL-C and chronic pain were found in the subgroup analysis.

**Conclusion:**

This study demonstrates a negative association between HDL-C levels and chronic pain in US adults, providing insights into the pathogenesis of chronic pain and potential improvements in chronic pain management strategies.

## 1 Introduction

Chronic pain, characterized as pain that lingers beyond the typical healing time frame (usually 3 months), poses a significant burden on individuals and economies (1). Research indicates that over 30% of people worldwide are affected by chronic pain (2). Individuals with chronic pain are more likely to experience anxiety, depression, activity limitations, opioid dependence, and reduced quality of life (3–5). Currently, the mechanisms underlying chronic pain remain challenging to identify, resulting in suboptimal management strategies that focus solely on symptoms or diseases (2). Therefore, to develop better prevention and management strategies, more research into risk factors for chronic pain is needed (6).

Chronic pain is typically viewed as a symptom rather than a disease. A variety of conditions, such as cardiovascular disease, diabetes, arthritis, and disk disease, may present with symptoms of chronic pain (7). Lipids are crucially involved in the pathophysiological processes of chronic pain. High-density lipoprotein cholesterol (HDL-C) is a molecule that is prevalent in or bound to HDL (8). HDL-C is considered beneficial for human health, and an increasing body of evidence suggests that low levels of HDL-C are associated with the aforementioned conditions and their concomitant symptoms of chronic pain, such as chest pain associated with acute coronary disease (9), neuropathic pain linked to diabetic peripheral neuropathy in individuals with type 2 diabetes (10), pain from osteoarthritis (11), and back pain associated with disc degeneration (12). HDL-C mediates anti-inflammatory and antioxidant effects (13). Low levels of HDL-C can lead to endothelial dysfunction, oxidative stress (10), and abnormal cytokine production, all of which are implicated in the pathogenesis of chronic pain (2). Therefore, we hypothesize that HDL-C may be associated with chronic pain.

Although several cohort studies in other countries have presented conflicting findings (14–17), there have been no reports on the independent association between HDL-C levels and chronic pain in American adults. As a result, the objective of this study is to explore this relationship utilizing data derived from the 2003–2004 National Health and Nutrition Examination Survey (NHANES).

## 2 Materials and methods

### 2.1 Study population

The study was conducted using NHANES data from 2003 to 2004. The NHANES project, a national survey spearheaded by the National Center for Health Statistics (NCHS) under the Centers for Disease Control and Prevention (CDC), is designed to evaluate the health and nutritional conditions of non-institutionalized US civilians. Utilizing a stratified multi-stage probability sampling approach, this survey has been in operation since 1999. It gathers nationally representative data through interviews covering demographic, dietary, socioeconomic, and health-related issues, as well as physical examinations and laboratory tests. The survey is conducted every 2 years. The NHANES database includes three consecutive cycles of chronic pain data (1999–2000, 2001–2002, and 2003–2004). The 1999–2002 HDL cholesterol measurements showed unsatisfactory deviations (> 4%) from the laboratory quality control (Solomon Park Research Laboratory, Kirkland, Washington), which is determined by the CDC. Therefore, NHANES data from 2003–2004 was chosen as the bias of the HDL-C method was acceptable (<4%).

### 2.2 Chronic pain

The NHANES database only conducted the Miscellaneous Pain section of the questionnaire survey for adults over the age of 20. Chronic pain was defined using the variables MPQ100 (pain duration exceeding 24 h in the last month) and MPQ110 (pain duration). Participants experiencing pain issues for a duration of 3 months or more were categorized into the chronic pain group (18). On the other hand, participants who indicated no pain issues in the previous month, as well as those with pain issues lasting less than 3 months, were grouped into the non-chronic pain category.

### 2.3 HDL-cholesterol

HDL-cholesterol was measured using the direct immunoassay method. Blood samples were processed, stored, and shipped to Johns Hopkins Hospital in Baltimore, Maryland for analysis. The NHANES Quality Assurance and Quality Control protocol adheres to the Clinical Laboratory Improvement Amendments Act of 1988. The NHANES Laboratory/Medical Technologist Procedure Manual provides detailed instructions on specimen collection, processing, quality control, and quality assurance.

### 2.4 Other covariates

Several potential covariates extracted from the NHANES were assessed, including age, sex, body mass index (BMI), marital status (living alone and married or living with a partner), alcohol consumption (average number of drinks per occasion with alcohol in the past year), smoking status (19), poverty income ratio (PIR), education level (did not graduate from high school, graduated from high school, and college education or above), race (Mexican American, other Hispanic non-Hispanic white non-Hispanic black, and other races), physical activity, triglyceride (TG), total cholesterol (TC), cotinine, blood lead, arthritis, cancer or malignancy, osteoporosis, hypertension, diabetes, and coronary heart disease. Physical activity was self-reported and categorized into four groups: mainly sit, walk around, light load, and heavy load. Hypertension and diabetes mellitus were determined based on medication use, self-reported physician diagnosis, and relevant testing indicators including blood pressure measurements, fasting blood glucose levels, and hemoglobin A1c levels (20, 21).

### 2.5 Statistical analysis

Data analyses were conducted using the statistical software packages R and Free Statistics software version 1.7.1 (22). Descriptive statistics were employed to summarize all data, encapsulating both the frequencies (percentages) and the mean ± standard deviation. The Chi-square test was used to evaluate differences between groups for categorical variables, while the Student’s *t*-test or the Mann-Whitney U-test was used for continuous variables, depending on which was most suitable. Multivariate logistic regression was conducted to explore the association between HDL-C levels and the risk of chronic pain. Four models were used for the multivariate logistic regression analysis, adjusting for various sociodemographic and clinical covariates. Model 1 was calibrated with age, sex, PIR, education level, marital status, and race. Model 2 incorporated all covariates from Model 1, with the addition of smoking status, alcohol consumption, physical activity, body mass index, coronary heart disease, arthritis, cancer or malignancy, osteoporosis, diabetes, and hypertension. Model 3 was an extension of Model 2, further adjusted for total cholesterol, triglyceride, cotinine, and blood lead. The likelihood ratio test was conducted on the interaction between subgroups, and sensitivity analysis was performed after excluding participants with missing data.

## 3 Results

### 3.1 Participants characteristics

**FIGURE 1:**
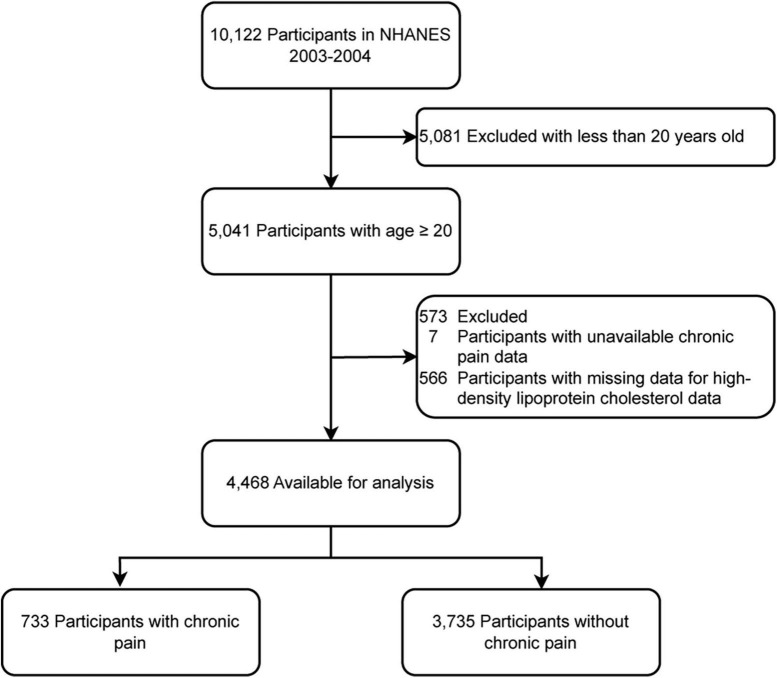
Study flow chart.

A total of 10,122 potential participants were identified, and 4,468 adults (≥ 20 years) were included in the study. The flowchart illustrating the exclusion criteria is presented in Figure 1. The baseline characteristics of patients according to HDL-C level categories are shown in Table 1. Table 1 shows that individuals with higher levels of HDL-C were more likely to have a lower risk of chronic pain.

**TABLE 1:** Characteristics of participants.

Variables	HDL-C, mg/dL					
	Total	Q1 (<43)	Q2 (43–51)	Q3 (52–63)	Q4 (≥64)	*P*-value
NO.	4468	1083	1080	1142	1163	
Chronic pain, n (%)	733 (16.4)	195 (18)	195 (18.1)	180 (15.8)	163 (14)	0.025
Age, year	50.5 ± 19.5	50.0 ± 18.5	50.9 ± 19.2	50.2 ± 19.6	50.8 ± 20.4	0.648
Sex, male, n (%)	2168 (48.5)	764 (70.5)	624 (57.8)	476 (41.7)	304 (26.1)	<0.001
Body mass index, kg/m^2^	28.4 ± 6.2	30.4 ± 6.1	29.1 ± 6.2	27.9 ± 5.9	26.4 ± 5.9	<0.001
**Marital status, n (%)**						<0.001
Married or living with a partner	2709 (60.7)	698 (64.6)	675 (62.6)	680 (59.5)	656 (56.4)	
Living alone	1756 (39.3)	383 (35.4)	404 (37.4)	462 (40.5)	507 (43.6)	
**Race, n (%)**						<0.001
Mexican American	896 (20.1)	247 (22.8)	237 (21.9)	227 (19.9)	185 (15.9)	
Other Hispanic	138 (3.1)	38 (3.5)	37 (3.4)	35 (3.1)	28 (2.4)	
Non-Hispanic white	2385 (53.4)	592 (54.7)	563 (52.1)	599 (52.5)	631 (54.3)	
Non-Hispanic black	858 (19.2)	161 (14.9)	191 (17.7)	237 (20.8)	269 (23.1)	
Other races	191 (4.3)	45 (4.2)	52 (4.8)	44 (3.9)	50 (4.3)	
**Education level, n (%)**						<0.001
Did not graduate from high school	1301 (29.2)	328 (30.3)	334 (31)	344 (30.2)	295 (25.4)	
Graduated from high school	1123 (25.2)	313 (28.9)	274 (25.4)	265 (23.2)	271 (23.3)	
College education or above	2037 (45.7)	442 (40.8)	469 (43.5)	531 (46.6)	595 (51.2)	
Poverty income ratio	2.2 (1.2, 4.0)	2.0 (1.1, 3.8)	2.2 (1.2, 4.0)	2.2 (1.3, 4.1)	2.2 (1.2, 4.3)	0.004
Smoking status, ≥ 100 cigarettes in life, n (%)	2230 (49.9)	624 (57.6)	561 (52)	536 (47)	509 (43.8)	<0.001
Alcohol consumption, average number of drinks	1.0 (0.0, 3.0)	1.0 (0.0, 3.0)	1.0 (0.0, 3.0)	1.0 (0.0, 3.0)	1.0 (1.0, 2.0)	0.156
**Physical activities, n (%)**						0.017
Mainly sit	1148 (25.7)	297 (27.4)	282 (26.1)	267 (23.4)	302 (26)	
Walk around	2337 (52.4)	542 (50.1)	567 (52.5)	599 (52.5)	629 (54.1)	
Light load	672 (15.1)	152 (14)	160 (14.8)	185 (16.2)	175 (15.1)	
Heavy load	307 (6.9)	91 (8.4)	70 (6.5)	90 (7.9)	56 (4.8)	
Triglyceride, mg/dL	111.5 (75.0, 167.0)	168.0 (114.0, 253.5)	123.0 (87.0, 173.0)	98.0 (69.0, 135.0)	82.0 (59.0, 119.5)	<0.001
Total cholesterol, mg/dL	202.1 ± 44.1	196.9 ± 48.2	199.6 ± 42.3	199.9 ± 43.8	211.6 ± 40.3	<0.001
Cotinine, ng/mL	0.1 (0.0, 23.4)	0.2 (0.0, 123.5)	0.1 (0.0, 44.6)	0.1 (0.0, 2.6)	0.1 (0.0, 0.9)	<0.001
Blood lead, ug/dL	1.6 (1.1, 2.6)	1.7 (1.2, 2.6)	1.7 (1.2, 2.6)	1.6 (1.0, 2.5)	1.5 (1.0, 2.5)	<0.001
Arthritis, n (%)	1250 (28.1)	301 (27.9)	294 (27.3)	332 (29.2)	323 (27.8)	0.789
Coronary heart disease, n (%)	225 (5.1)	69 (6.4)	70 (6.5)	53 (4.7)	33 (2.8)	<0.001
Cancer or malignancy, n (%)	410 (9.2)	89 (8.2)	117 (10.9)	95 (8.3)	109 (9.4)	0.12
Osteoporosis, n (%)	323 (7.2)	58 (5.4)	57 (5.3)	95 (8.3)	114 (9.8)	<0.001
Hypertension, n (%)	1810 (40.5)	460 (42.5)	469 (43.4)	440 (38.5)	441 (37.9)	0.014
Diabetes, n (%)	615 (13.8)	218 (20.1)	168 (15.6)	127 (11.1)	102 (8.8)	<0.001

Data are shown as mean ± SD, median (IQR), or n (%). NHANES, National Health and Nutrition Examination Survey; NO., number; Q, quartile; HDL-C, high-density lipoprotein cholesterol.

### 3.2 Relationship between HDL-C and chronic pain

Univariate analysis revealed that age, sex, physical activity, PIR, BMI, smoking status, alcohol consumption, triglyceride levels, cotinine levels, arthritis, cancer or malignancy, coronary heart disease, osteoporosis, hypertension, and diabetes were associated with chronic pain (Table 2).

**TABLE 2:** Association of covariates with chronic pain risk.

Variable	OR (95% CI)	*P*-value
Age, year	1.01 (1∼1.01)	<0.001
**Sex, n**		
Male	Ref.	
Female	1.38 (1.17∼1.62)	<0.001
Body mass index, kg/m2	1.02 (1.01∼1.04)	<0.001
**Marital status, n**		
Married or living with a partner	Ref.	
Living alone	1 (0.85∼1.17)	0.973
**Race, n**		
Mexican American	Ref.	
Other Hispanic	0.91 (0.5∼1.64)	0.751
Non-Hispanic white	1.97 (1.56∼2.48)	<0.001
Non-Hispanic black	1.27 (0.96∼1.69)	0.100
Other races	1.81 (1.18∼2.75)	0.006
**Education level, n**		
Did not graduate from high school	Ref.	
Graduated from high school	1.06 (0.86∼1.31)	0.584
College education or above	0.89 (0.73∼1.07)	0.211
Poverty income ratio	0.91 (0.86∼0.95)	<0.001
**Smoking status, 100 cigarettes in life, n**		
No	Ref.	
Yes	0.64 (0.54∼0.75)	<0.001
Alcohol consumption, average number of drinks	0.96 (0.92∼0.99)	0.018
**Physical activities, n**		
Mainly sit	Ref.	
Walk around	0.66 (0.55∼0.79)	<0.001
Light load	0.77 (0.6∼0.99)	0.039
Heavy load	0.64 (0.45∼0.91)	0.014
High-density lipoprotein cholesterol, mg/dL	0.99 (0.99∼1)	<0.001
Total cholesterol, per 10 mg/dL	1.02 (1∼1.03)	0.077
Triglyceride, per 10 mg/dL	1.01 (1∼1.01)	0.009
Cotinine, per 10 ng/mL	1.01 (1.01∼1.02)	<0.001
Blood lead, ug/dL	1.03 (0.99∼1.07)	0.111
**Arthritis, n**		
No	Ref.	
Yes	3.83 (3.25∼4.51)	<0.001
**Cancer or malignancy, n**		
No	Ref.	
Yes	1.72 (1.35∼2.19)	<0.001
**Osteoporosis, n**		
No	Ref.	
Yes	2.59 (2.02∼3.32)	<0.001
**Hypertension, n**		
No	Ref.	
Yes	1.67 (1.42∼1.96)	<0.001
**Diabetes, n**		
No	Ref.	
Yes	1.39 (1.13∼1.73)	0.002
**Coronary heart disease, n**		
No	Ref.	
Yes	2.1 (1.55∼2.83)	<0.001

OR, odds ratio; CI, confidence interval; Ref, reference.

Table 3 presents the association between HDL-C and chronic pain. In Model 1, adjusting for age, sex, marital status, PIR, race, and education level, HDL-C was negatively related to chronic pain (HDL-C per 20 unit, OR: 0.74 [0.66–0.83], *P* < 0.001). This association remained stable even after adjusting for additional potential covariates in Model 2–3 [HDL-C per 20 mg/L; Model 2: OR = 0.79 (0.7–0.89), *P* < 0.001; Model 3: OR = 0.74 (0.66–0.83)]. Additionally, in the fully adjusted model, compared with individuals in the lowest HDL-C level (mg/dL) group (Q1, <43), the odds ratios (ORs) for chronic pain in the Q2 (43-51), Q3 (52–63), and Q4 (≥64) groups were 1.06 (95% CI: 0.83–1.35), 0.89 (95% CI: 0.69–1.15), and 0.76 (95% CI: 0.58–1.01), respectively. Compared with the Q1 group, there was no significant change in the risk of chronic pain in the Q2 group, while in the Q3–4 groups, the risk of chronic pain was reduced by 11% and 24%, respectively.

**TABLE 3:** Association of high-density lipoprotein cholesterol with chronic pain.

Variable	Event, n (%)	Crude Model		Model 1		Model 2		Model 3	
		OR (95% CI)	*P-*value	OR (95% CI)	*P-*value	OR (95% CI)	*P-*value	OR (95% CI)	*P-*value
HDL-C, per 20 mg/dL	733/5034 (16.4)	0.82 (0.74∼0.91)	<0.001	0.74 (0.66∼0.83)	<0.001	0.79 (0.7∼0.89)	<0.001	0.74 (0.66∼0.83)	<0.001
**HDL-C, quartile**									
<43	195/1083 (18)	1(Reference)		1(Reference)		1(Reference)		1(Reference)	
43–51	195/1080 (18.1)	1 (0.81∼1.25)	0.976	0.96 (0.77∼1.21)	0.751	1.04 (0.82∼1.32)	0.73	1.06 (0.83∼1.35)	0.653
52–63	180/1142 (15.8)	0.85 (0.68∼1.06)	0.158	0.76 (0.61∼0.96)	0.024	0.86 (0.67∼1.1)	0.23	0.89 (0.69∼1.15)	0.361
≥64	163/1163 (14)	0.74 (0.59∼0.93)	0.01	0.61 (0.48∼0.78)	<0.001	0.74 (0.57∼0.97)	0.028	0.76 (0.58∼1.01)	0.058
*P* for trend			0.004		<0.001		0.012		0.028

OR, odds ratio; CI, confidence interval. Model 1 was adjusted for age, sex, marital status, poverty income ratio, race, and education level. Model 2 was adjusted for Model 1 + alcohol consumption, smoking status, physical activity, body mass index, coronary heart disease, arthritis, cancer or malignancy, osteoporosis, diabetes, and hypertension. Model 3 was adjusted for Model 2 + total cholesterol, triglyceride, cotinine, and blood lead.

### 3.3 Sensitivity analysis

**FIGURE 2:**
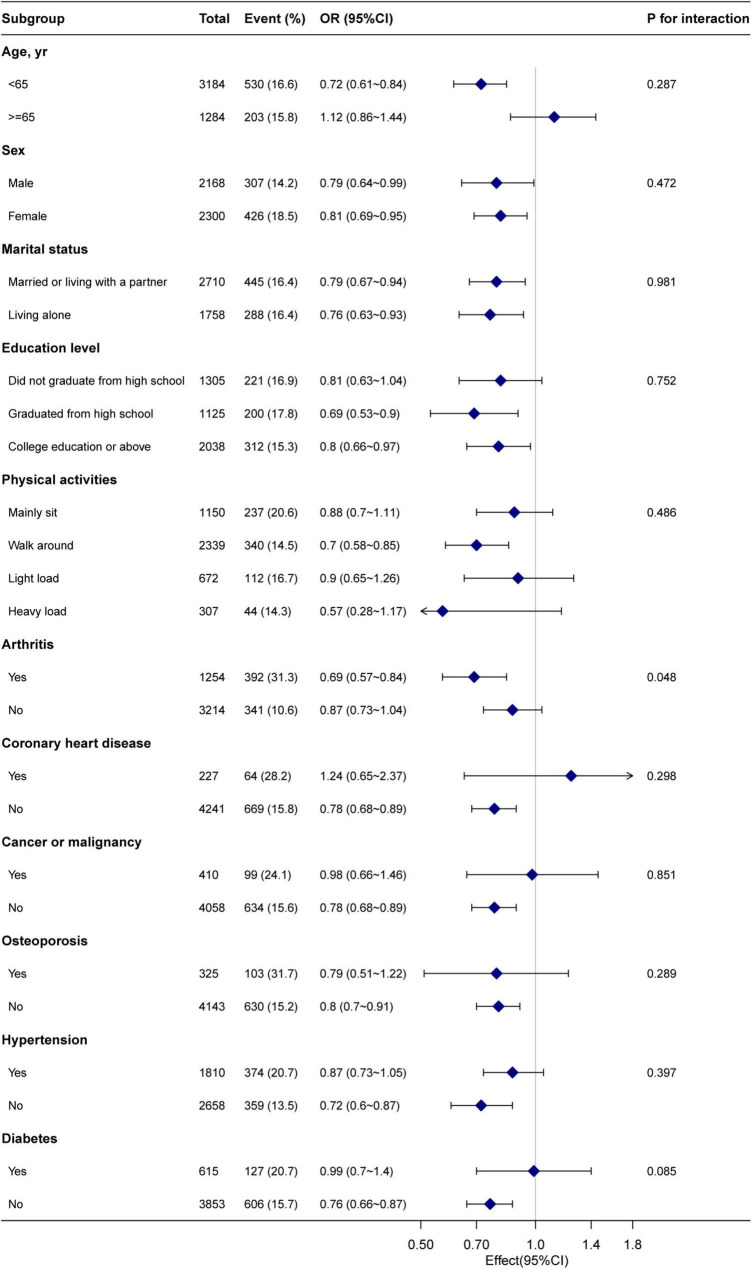
Subgroup analysis of the high-density lipoprotein cholesterol and chronic pain. Each stratification factor was adjusted for age, sex, body mass index, marital status, poverty income ratio, race, alcohol consumption, smoking status, education level, physical activity, coronary heart disease, arthritis, cancer or malignancy, osteoporosis, hypertension, diabetes, triglyceride, total cholesterol, cotinine, and blood lead.

Figure 2 shows that no significant interactions were observed after stratification by sex, age (< 65 and ≥65 years), marital status, education level, physical activity, arthritis, coronary heart disease, cancer or malignancy, osteoporosis, hypertension, and diabetes. Due to multiple testing, the *P*-value (0.048) of the interaction in the arthritis subgroup may not be statistically significant. After excluding individuals with missing data (leaving 3,205 participants), the relationship between HDL-C and chronic pain remained robust in the sensitivity analysis (Table 4).

**TABLE 4:** Association of high-density lipoprotein cholesterol with chronic pain (3,205 participants with complete data).

Variable	Event, n (%)	Crude Model		Model 2		Model 3		Model 4	
		OR (95% CI)	*P*-value	OR (95% CI)	*P*-value	OR (95% CI)	*P*-value	OR (95% CI)	*P*-value
HDL-C, per 20 mg/dL	542/3205 (16.9)	0.84 (0.74∼0.95)	0.004	0.75 (0.66∼0.86)	<0.001	0.81 (0.71∼0.94)	0.005	0.8 (0.69∼0.93)	0.004
**HDL-C, quartile**									
<43	144/772 (18.7)	1(Reference)		1(Reference)		1(Reference)		1(Reference)	
43–51	144/784 (18.4)	0.98 (0.76∼1.27)	0.885	0.96 (0.73∼1.25)	0.742	1.02 (0.77∼1.34)	0.9	1.02 (0.77∼1.35)	0.896
52–63	130/823 (15.8)	0.82 (0.63∼1.06)	0.131	0.75 (0.57∼0.99)	0.044	0.84 (0.62∼1.12)	0.232	0.85 (0.63∼1.15)	0.293
≥64	124/826 (15)	0.77 (0.59∼1)	0.052	0.64 (0.48∼0.85)	0.002	0.78 (0.57∼1.07)	0.124	0.77 (0.56∼1.08)	0.129
*P* for trend			0.023		0.001		0.069		0.077

OR, odds ratio; CI, confidence interval. Model 1 was adjusted for age, sex, marital status, poverty income ratio, race, and education level. Model 2 was adjusted for Model 1 + alcohol consumption, smoking status, physical activity, body mass index, coronary heart disease, arthritis, cancer or malignancy, osteoporosis, diabetes, and hypertension. Model 3 was adjusted for Model 2 + total cholesterol, triglyceride, cotinine, and blood lead.

## 4 Discussion

This study utilized a nationally representative database from NHANES to investigate the relationship between HDL-C and chronic pain in US adults. The findings showed that as HDL-C increased, the incidence of chronic pain decreased. Specifically, for every 20 units increase in HDL-C level, the risk of chronic pain is reduced by 26%. The relationship between HDL-C and chronic pain remained consistent in both subgroups and sensitivity analyses.

Few studies have explored the relationship between chronic pain and HDL-C. A cohort study with a sample size of 13,328 from the Scottish Family Health Study found that after adjustment by age and sex, HDL-C levels were associated with chronic pain [OR, 0.69 (0.63–0.76)]. Our findings are similar to this study, but our analysis also adjusts for additional conditions that may contribute to chronic pain, such as arthritis. Other studies examining specific types of chronic pain and HDL-C levels have yielded different conclusions. For example, studies on women with fibromyalgia (14) and shoulder and neck pain (23) found no association with HDL-C, while studies on chronic low back pain showed a negative correlation with HDL-C (17, 23). These discrepancies may be attributed to the different pathogenic mechanisms underlying different types of chronic pain.

Chronic pain can arise from nociceptive stimuli (caused by tissue injury), neuropathic stimuli (caused by nerve injury), or nociplastic stimuli (caused by a sensitized nervous system). It can also result from a combination of these factors (2). Currently, there is no established mechanism to explain the relationship between chronic pain and HDL-C, but we have proposed several possible mechanisms. Firstly, injurious pain may occur due to tissue damage or degenerative changes, such as those seen in inflammatory diseases like primary osteoarthritis and traumatic arthritis (2). Inflammatory responses have been associated with decreased levels of HDL-C (24, 25). Secondly, neuropathic pain can be caused by diseases or injuries that affect the nervous system, such as nerve compression, metabolism issues, or ischemia (2). For instance, in diabetes, low HDL-C levels may contribute to endothelial dysfunction, oxidative stress, and abnormal cytokine production (26), which are linked to the development of painful diabetic neuropathy (27, 28).

Given the high prevalence and significant impact of chronic pain, it is essential to understand its risk factors. This study contributes to the existing literature by examining the correlation between HDL-C and chronic pain. Although HDL-C is not currently a target for drug treatment (29), lifestyle changes such as physical activity, weight loss, alcohol consumption, smoking cessation, and adopting a Mediterranean diet have been shown to increase HDL-C levels (30). However, considering the limited mobility and physical or functional impairment often experienced by patients with chronic pain, lifestyle changes to increase HDL-C levels should be individualized.

The strength of this study lies in its ability to provide new evidence on the relationship between HDL-C levels and chronic pain. The findings are particularly compelling due to the extensive use of a large, representative US cohort, rigorous quality control measures, and the application of multiple statistical methods that consistently produced stable results.

However, the present study is subject to certain limitations. Firstly, given the observational nature of this study, a causal relationship between HDL-C and chronic pain cannot be conclusively established. Secondly, the findings of this research are based solely on data obtained from the NHANES database, which represents American adults. Consequently, additional investigations are necessary to ascertain the generalizability of these results to other populations. Lastly, the absence of repeated measurements of HDL-C data may compromise the accuracy of its long-term levels and its association with chronic pain.

## 5 Conclusion

There is a negative relationship between HDL-C and chronic pain, with individuals having low HDL-C levels being at a higher risk of chronic pain. Furthermore, this study posits that HDL-C potentially influences the development of chronic pain and could enhance the efficacy of chronic pain management approaches.

## Data availability statement

The raw data supporting the conclusions of this article will be made available by the authors, without undue reservation.

## Ethics statement

The studies involving humans were approved by The National Center for Health Statistics Research Ethics Review Committee. The studies were conducted in accordance with the local legislation and institutional requirements. The participants provided their written informed consent to participate in this study.

## Author contributions

HW: Writing–review and editing, Supervision, Conceptualization. PM: Data curation, Writing–original draft, Methodology, Formal analysis. HD: Data curation, Writing–original draft, Methodology, Formal analysis. SC: Writing–original draft, Formal analysis, Data curation. XG: Writing–original draft, Formal analysis, Data curation. XC: Writing–original draft, Formal analysis, Data curation. YL: Writing–original draft, Formal analysis, Data curation. GF: Writing–review and editing, Supervision, Project administration, Conceptualization.
